# Comparative metabolomics analysis reveals dynamic changes in carbohydrate profiles of corms during the “relay growth” of konjac (*Amorphophallus muelleri*)

**DOI:** 10.3389/fpls.2023.1259561

**Published:** 2023-10-18

**Authors:** Ying Qi, Penghua Gao, Shaowu Yang, Lifang Li, Yanguo Ke, Huanyu Wei, Feiyan Huang, Lei Yu

**Affiliations:** College of Agronomy, Yunnan Urban Agricultural Engineering and Technological Research Center, Kunming University, Kunming, China

**Keywords:** konjac glucomannan, carbohydrates, corms turnover, sucrose, starch, *Amorphophallus muelleri*

## Abstract

The type and content of carbohydrates in konjac corms are an essential factors in determining the quality of konjac; however, the pattern of carbohydrate changes and the mechanism regulating the development of mother and daughter corms in the “relay growth” process of *Amorphophallus muelleri* remain unclear. This study aimed to investigate changes in corm carbohydrates during the growth cycle of *A. muelleri* and to compare the carbohydrate composition and the expression of related genes between mother and daughter corms. Integrated metabolome and RNA-seq analyses identified 37 differential metabolites as well as 8074 genes that were differentially expressed between mother and daughter corms, the majority of which were involved in starch and sucrose metabolism. More than 80% of the differential metabolites, including sucrose and starch, tended to accumulate in the mother corms; however, konjac glucomannan (KGM), as one of the most important carbohydrates and its major component of the corm, accumulated in higher amounts in the daughter corms. In addition, the expression of invertase and alpha-amylase that promote the breakdown of sucrose and starch was 351.78- and 15.63-fold higher, respectively, in the daughter corm, whereas that of the starch synthesis gene *AkWAXY* was only 0.096 times as high as in the mother corms. Furthermore, the level of cellulose synthase-like protein G, which promotes KGM synthesis, was 3.85 times higher in daughter corms compared to mother corms. Thus, we inferred that the daughter and mother corms had two distinct carbohydrate utilization strategies. This study provides insights into temporal changes in carbohydrates during the growth cycle of *A. muelleri*.

## 1 Introduction

Konjac, a collective name for a subset of the genus *Amorphophallus*, family Araceae (Claudel et al., 2017), are perennial geophytes that have been cultivated in China for more than 3,000 years (Zhang et al., 1999). Records on the consumption and medicinal use of konjac can be traced back to the Han Dynasty (Zhang and Wang, 2010; Pouchon et al., 2023). The corm of konjac is the most valuable organ of the plant, with carbohydrates being its core component (Liu, 2004). Carbohydrate content, structure, and composition are critical factors directly affecting its quality and economic value (Yu et al., 2022). Konjac corm contains a variety of carbohydrates, the most noteworthy of which is glucomannan. Konjac glucomannan (KGM) is a natural polysaccharide with strong water absorption, high swelling rate, and good gelling and film-forming properties; therefore, it has important applications in medical, agricultural, chemical, and other industries (Zalewski et al., 2015; Yang et al., 2017; Devaraj et al., 2018; Chen et al., 2023). Plants with non-starch polysaccharides as the main storage material exist only in some genera, and konjac is currently among the few plant taxa with glucomannan as the main storage carbohydrate. Consequently, it is necessary to explore the molecular mechanisms underlying the corm carbohydrate accumulation process (Khan and Marya, 2019).

The growth pattern of *A. muelleri* is distinctly different from that of *A. konjac* (the current main cultivar in Asia), which bears a single leaf per growing season, with the corm expanding approximately four to six times per season (Hetterscheid et al., 2020). In contrast, *A. muelleri* has the characteristics of multiple seedling “relay” growth, where multiple leaves grow sequentially and collectively feed one corm in a growth cycle. Its leafy corms can expand at a rate of more than 100 times in one season (Zhang and Wang, 2010). This multileaf growth feature increases the leaf area exposed to light, promoting the synthesis and accumulation of organic carbon in the leaves, and may contribute significantly to the high corm expansion rate. However, the changes in carbohydrates in corms during the successive growth of leaves are unclear. As leaves grow, the mother corm gradually shrinks, and as the first leaf reaches maturity, the base of the petiole swells to form the daughter corm. At the second leaf sprouting stage, the new larger daughter corm replaces the mother corm, thereby completing the turnover process (Xue et al., 2022).

The konjac corm is an important storage organ, and the growth of new tissues depends heavily on stored nutrients. As the resources available to plants are limited, there are trade-offs in using growth resources among individual organs (Ruan et al., 2013; Bénard et al., 2015). The mother corm can act as a source of carbohydrates and other nutrients to maintain plant growth during periods of low photosynthesis, such as dormancy and germination (Villordon et al., 2014; Akoumianakis et al., 2016). As the leaf matures, its photosynthetic capacity increases, and the corm can, in turn, act as a sink to receive products from the photosynthetic organ and store them in the form of carbohydrates (Yu et al., 2022). The “relay” growth process of *A. muelleri* involves the formation of multiple leaves and new corms, as well as the recycling of nutrients after leaf senescence, which is a complex physiological process. As an energy source and a component of the cytoskeleton, carbohydrates play an essential role in regulating the tuberization process (Araya et al., 2006; Aguirre et al., 2018). However, it is unclear whether there are differences in the composition and distribution of carbohydrates and carbohydrate-based photosynthetic products between mother and daughter corms. In this study, we detected dynamic changes in corm carbohydrates throughout the growth cycle of *A. muelleri*, and compared the carbohydrate composition and the expression of related genes between the mother and daughter corms. For *A. muelleri*, KGM is the main carbohydrate and component in the corm; for the products whose main purpose is to obtain KGM, the content and purity of KGM are the most important indicators to determine the quality. This is the first study investigating the accumulation of KGM during corm turnover in konjac, where energy is mainly stored in the form of non-starch polysaccharides. Understanding this process is essential to devise strategies for the improvement of konjac yield and quality.

## 2 Materials and methods

### 2.1 Plant materials and determination of growth traits

Two-year-old corms of *A. muelleri*, uniform in size and weight (approximately 100 g), were selected and grown in a greenhouse at the Yunnan Urban Agricultural Engineering and Technological Research Center, Kunming University (24.98°N, 102.79°E; altitude: 1934 m) from May–November 2020. Each corm was weighed and labeled before being transplanted into 10 L plastic pots containing 8 kg substrate (white sod peat: white peat: peat fibers = 6:3:1; pH value: 6.0; NPK fertilizer level: 1.0 g L^-1^; Klasmann, Germany). At the beginning of the growth phase, greenhouse conditions were maintained as follows: 25–35°C, shade conditions< 50%, and a relative humidity of 60–80%. Water-soluble fertilizer is 20% of the total N, 20% of P_2_O_5_, 20% of K_2_O, and B + Zn + Mn content ≥ 0.2% (Tianteng, YunTianHua Co., LTD, China). Watering and fertilization were performed as needed.

**Figure 1:**
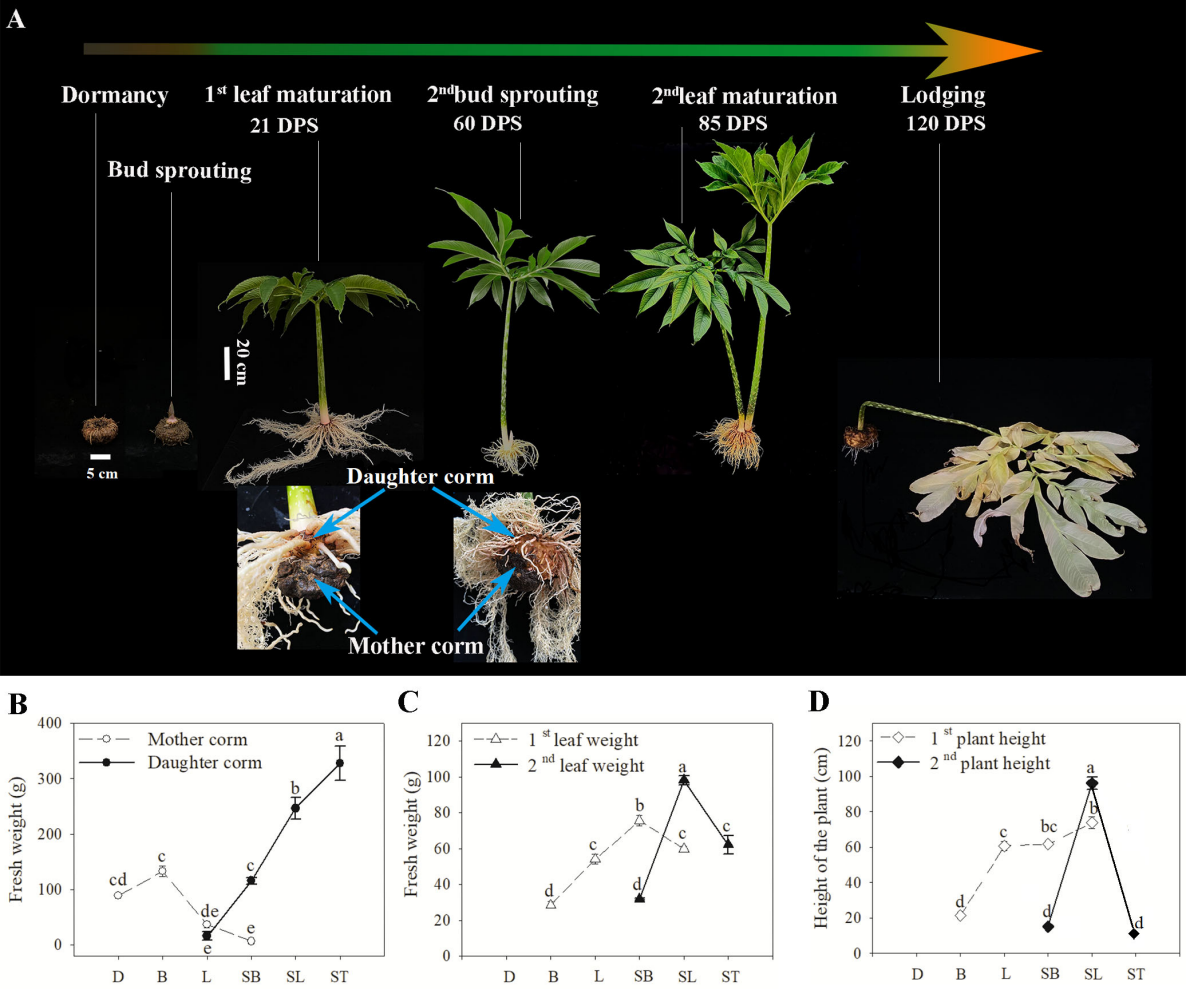
**(A)** A diagram illustrating the growth cycle and growth traits of *A muelleri*. Eighteen samples from six key developmental stages were collected for metabolic profiling and RNA-seq at the stages indicated. DPS represents the number of days post sprouting. **(B-D)** Growth characteristics of *A muelleri* measured in this study. The axis indicates the sample harvest date. The changes in fresh weight of corms **(B)** and leaves **(C)**, and plant height **(D)** of *A muelleri* during the growth cycle are shown. D, dormant corms; B, bud sprouting stage; L, first leaf maturation stage; SB, second bud sprouting stage; SL, second leaf maturation stage; ST, lodging period. Each data point represents the mean ± SE (n ≥ 6). The different letters above the bars indicate significant differences between developmental periods (P<0.05; based on One-way ANOVA followed by a *post-hoc* Tukey’s test).

As shown in **Figure 1A**, dormant corms (D) were those stored at 20°C for 2 weeks without sprouting (Campbell et al., 2010). Corms with buds that had germinated 5–8 cm were used as material for the bud sprouting stage (B). At the time of full expansion of the first leaf, the mother corm was used as material for the first leaf maturation stage (L). Sampling of the daughter corms during the second bud sprouting stage (SB) was performed when the second buds were 5–8 cm long. The daughter corm at the time of full expansion of the second leaf was used as material for the second leaf maturation stage (SL). When the second leaves withered and wilted, the corm was in the lodging period (ST). Growth traits, including the fresh weight of corms and leaves (excluding petioles) and the plant height (the shortest distance from the base of the petiole to the highest point of the leaf), were measured at each period. At least six individuals per period were selected for the measurement.

### 2.2 Measurements of glucomannan and starch content of *Amorphophallus muelleri*

After removing the bud and skin, corms were cut into 1-mm thick slices and dried at 80°C for 48 h. After grinding, a powder with a particle diameter between 0.125 mm and 0.178 mm was reserved to measure KGM and starch content. Samples from three individual plants were mixed to form one biological replicate, and three biological replicates were used for KGM and starch measurements.

The glucomannan content of konjac was measured using the 3,5-dinitrosalicylic acid colorimetric method (Wang et al., 1998). Approximately 0.1 g of konjac powder was added to 25 mL of 0.1 M formic acid sodium hydroxide buffer and left stirring at 30°C to dissolve and swell for 4 h. The volume was fixed to 50 mL and centrifuged at 4000 rpm for 20 min. Then, 5 mL of extract solution (supernatant) was added to 2.5 mL of 3 M sulfuric acid, sealed and kept in a boiling water bath for 1.5 h. After cooling, 2.5 mL of 6 mol/L sodium hydroxide solution was added, and the volume was adjusted to 25 mL with distilled water; this solution was used for hydrolysis. Subsequently, 2 mL of extract was taken, hydrolysis solution and distilled water were added in a 25 mL volumetric flask, 1.5 mL of 1% 3,5-dinitrosalicylic acid was added and kept in a water bath for 5 min; the volume was adjusted with distilled water, and the absorbance value at 550 nm was measured spectrophotometrically (UV-2500, Shimadzu, Kyoto, Japan). Glucose was used for the standard curve, and distilled water served as a blank. Glucomannan content was calculated as follows:


$$\text{Konjac glucomannan content }\left(\%\right)=\frac{\in \left(5\text{T}-\text{T}0\right)\times 50}{\text{m}\times \left(1-\text{w}\right)\times 1000}\times 100$$

∈  is the ratio of the molecular weight of glucose and mannose produced by hydrolysis of glucomannan, calculated as 0.9; T = the weight of glucose (mg) in the glucomannan hydrolysate as found on the standard curve; T0 = the weight of glucose (mg) in the glucomannan extract solution as found on the standard curve; m = the weight of konjac powder sample (g); and w = the water content of konjac powder sample (%).

The starch content was measured using the anthrone colorimetric method (Guo and Peng, 2007). Approximately 0.1 g of konjac powder was placed in a centrifuge tube, 25 mL of hot 80% ethanol was added, mixed thoroughly, placed aside for 5 min, centrifuged at 2,500 rpm for 5 min, and the supernatant was removed. The remaining material was washed repeatedly using 80% ethanol. Approximately 5 mL of 52% perchloric acid solution was added to the precipitate, stirred for 10 min, centrifuged at 2,500 rpm for 10 min, and the supernatant was retained after filtration. The precipitate was dissolved in perchloric acid again, combined with the supernatant, and water was added to adjust the volume to 100 mL. Then, 1 mL of the extract was added to 3 mL of anthrone-sulfuric acid solution (0.4 g of anthrone dissolved in 100 mL of 80% sulfuric acid), kept in a water bath for 5 min, and absorbance was measured at 640 nm. Standard curves were generated using different concentrations of starch standard solutions, and samples of distilled water were used as a blank. The standard curve was plotted with absorbance on the x-axis and starch concentration on the y-axis, and the regression equation between absorbance and starch concentration was obtained by curve fitting.

### 2.3 LC-MS/MS-based metabolomic analysis

Samples for metabolite profile testing were collected from corms (approximately 5 mm below the buds) during the growth cycle. As the replacement of the old and new corms was completed by the second bud sprouting stage, samples from this stage to the lodging stage were collected from daughter corms, whereas samples from the dormant stage to the first leaf maturation stage were collected from mother corms. Samples from three individual plants were mixed to form each biological replicate, and three biological replicates were used for the metabolomics analysis.

The untargeted metabolomics assay was performed by Shanghai Applied Protein Technology Co., Ltd. (Shanghai, China). In brief, the samples were rapidly frozen, ground, and extracted using 1000 mL of methanol, acetonitrile, and water (2:2:1, v/v/v). The supernatant was dried in a vacuum centrifuge after centrifugation at 4°C, 14,000 g, for 15 min. The dried samples were then filtered through a SCAA-104 filter with a pore size of 0.22 μm (ANPEL, Shanghai, China) and dissolved in 100 μL of acetonitrile: water (1:1, v/v) prior to LC-MS analysis. Analyses were performed using an ultra-high performance liquid chromatograph (UHPLC; 1290 Infinity LC, Agilent Technologies, CA, USA) coupled to a quadrupole time-of-flight (AB Sciex TripleTOF 6600) system. A Waters ACQUITY UPLC CSH C18 column (150 x 2.1 mm, 1.7 mm, Waters Corporation, MA, USA) was used for UHPLC separation. Mobile phase A consisted of 25 mM ammonium acetate and 0.5% formic acid in water, whereas mobile phase B was composed of methanol. The temperature of the column was 40°C. The injection volume was 2 µL, and the flow rate was set to 0.4 mL/min. The sample was kept in an automated sampler at 4°C for the duration of the analysis. In order to track and assess the stability and dependability of the data, quality control (QC) samples were added to the sample queue. The apparatus was set up for automatic MS/MS acquisition with a m/z range of 25–1000 Da and a product ion scan accumulation time of 0.05 s/spectra. Using information-dependent acquisition in high-sensitivity mode, the product ion scan was recorded. This was performed according to the following criteria: exclude isotopes within 4 Da; collision energy: 35 V ± 15 eV; declustering potential: 60 V (+) and -60 V (-); candidate ions to monitor per cycle: 10.

**Figure 2:**
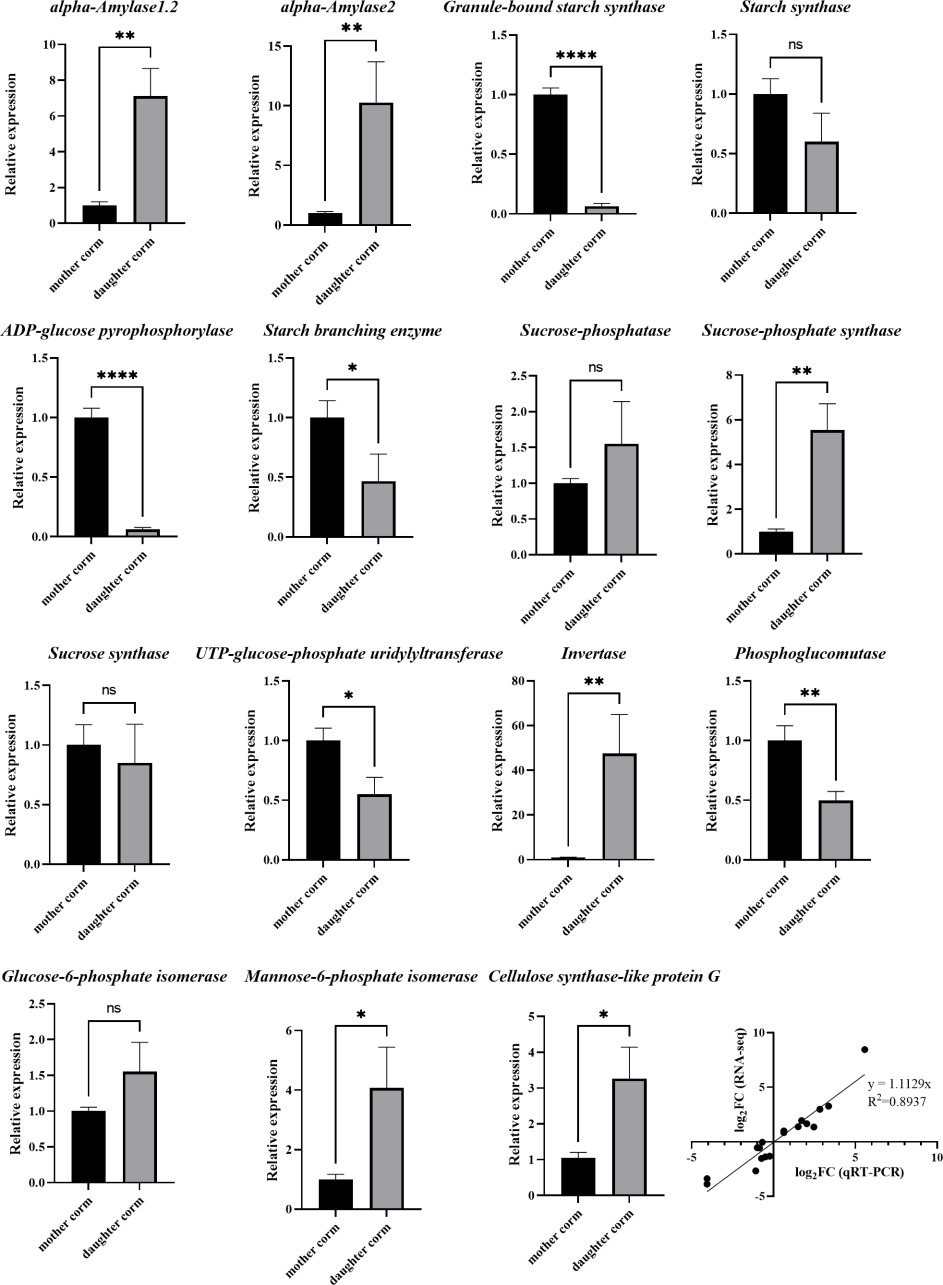
RT-qPCR analysis of genes involved in starch, sucrose and KGM biosynthesis compared between mother and daughter corms of *A. muelleri*. The data indicate mean values ± standard deviation of three biological replicates. Asterisks indicate statistical significance using Student’s t test (*: P< 0.05; **: P< 0.01; ****: P< 0.0001). The last graph is the Pearson correlation between the RNA-seq and the RT-qPCR.

The pretreatment of LC/MS raw data was performed by Progenesis QI (Waters Corporation, Milford, USA) software. Internal standard peaks, as well as any known false positive peaks (including noise, column bleed, and derivatized reagent peaks), were removed from the data matrix, duplicate peaks were eliminated, and then the remaining peaks were pooled together. **Figure 2** concurrently, the metabolites were identified by searching the database; the main databases were the HMDB (http://www.hmdb.ca/) and Metlin (https://metlin.scripps.edu/). The metabolites with VIP > 1, p< 0.05 were determined as significantly different.

### 2.4 RNA-sequencing analysis

Samples for RNA-seq were collected from the same locations (from the mother corms at the first leaf maturation stage and daughter corms at the second leaf maturation stage) as those used for metabolome analysis. Three biological replicates were included, each consisting of samples from at least three different plants.

Total RNA was extracted using a TRIzol reagent kit (Invitrogen, Carlsbad, CA, USA). The quality and concentration of the extracted RNA are shown in **Table S1**. Approximately 3 µg of RNA from each replicate was used to prepare the RNA-Seq library. Library preparation and RNA-seq analysis was performed by the Gene Denovo Biotechnology Company, and sequencing was performed using the Illumina Novaseq6000 platform (Guangzhou, China). Clean reads produced by the HiSeq X Ten sequencing platform were mapped to the konjac reference genome (https://www.ncbi.nlm.nih.gov/sra/PRJNA608095), and paired-end clean reads were mapped using HISAT2 (Kim et al., 2015). Using RSEM software, the fragments per kilobase of transcript per million mapped reads (FPKM) value was calculated to determine its expression abundance (Li and Dewey, 2011). A fold change (FC) of greater than two and an adjusted P value of less than 0.05 indicated significant up- or downregulation of genes.

### 2.5 RT–qPCR analysis

RNA extraction followed the description in the previous paragraph. First- strand cDNA synthesis followed the handbook of TUREscript 1^st^ Stand cDNA SYNTHESIS Kit (Aidlab, Beijing, China). Primers were designed by Beacon Designer 7.9, and specific information can be found in **Table S2**. RT–qPCR was performed using the qTOWER 2.0/2.2 Quantitative Real-Time PCR Thermal Cycler (Langewiesen, Germany) following the manufacturer’s instructions. The relative expression level of each gene was calculated using the ΔCt method. After detecting the transcript levels of eight housekeeping genes, including Actin, GAPDH, 18srRNA, EF-1β, QCR, β-Tubulin, CYP, and SAMDC, and using geNorm algorithm to determine the most stable reference gene (Mestdagh et al., 2009). Actin was selected as the internal reference gene.

### 2.6 Statistical analysis and plotting of data

After normalized to total peak intensity, the processed data were analyzed by the “ropls” package in R (version 3.4.4), where it was subjected to multivariate data analysis, including pareto-scaled principal component analysis (PCA). To compare the growth traits and the content of metabolites in the corms at different periods, we performed a One-way ANOVA followed by a *post-hoc* Tukey’s test using SPSS version 16.0 (SPSS Inc., USA), and P< 0.05 was considered significant. To compare the differences in metabolites between daughter and mother corms, Student’s t-test was used, and P< 0.05 was considered significant. Heatmaps were generated using TBtools (Chen et al., 2020), data were log_2_ scaled, and hierarchical clustering was performed using Pearson’s correlation distance/complete linkage method. Other line plots were generated using SigmaPlot version 10.0 (Systat Software Inc., USA).

## 3 Results and discussion

### 3.1 Changes in growth traits during the growth cycle of *Amorphophallus muelleri*

As shown in **Figure 1A**, both the above-ground leaves and below-ground corms undergo a turnover process in *A. muelleri*. With the development of leaves, the biomass of the mother corm declined noticeably (**Figure 1B**); by the time the first leaf matured, the fresh weight of the mother corm was only 36.92 g, less than one-third of the weight of the corm at the germination stage, while the fresh weight of the daughter corm at this time was 15.83 g, 42.88% of that of the mother corm at the same period. Subsequently, the daughter corm entered a period of rapid growth. At the second leaf emergence stage, the weight of the daughter corm was equal to the maximum weight of the mother corm, and it took approximately 25 days for the weight to increase to 246.49 g, which was nearly twice the maximum weight of the mother corm. The rapid expansion of daughter corms was accompanied by a drastic increase in the weight and height of the above-ground parts (**Figures 1C, D**). From the sprouting of the second leaf to maturity, the weight of the leaf increased from 31.75 g to 98.07 g, and the height of the plant increased from 15.07 cm to 96.20 cm. The second leaf was 1.30 times heavier and 1.56 times taller at maturity compared to the first leaf at the same stage. Details of the growth traits are presented in **Table S3**.

It took approximately 60 days from the bud sprouting stage (B) to the second leaf sprouting stage (SB) for the mother corm to convert. It took approximately 64 days from the first leaf maturation stage (L) to the second leaf maturation stage (SL) for leaves to be replaced. Therefore, the four periods, B, L, SB, and SL, are critical for the replacement of corms and leaves, and the corm is the main storage organ for photosynthetic products produced by the leaves. Thus, a comparison of corm material before and after conversion can reflect, at least to some extent, the differences in photosynthetic capacity between the two leaves grown in succession.

### 3.2 Differences in metabolites between mother and daughter corms

**Figure 3:**
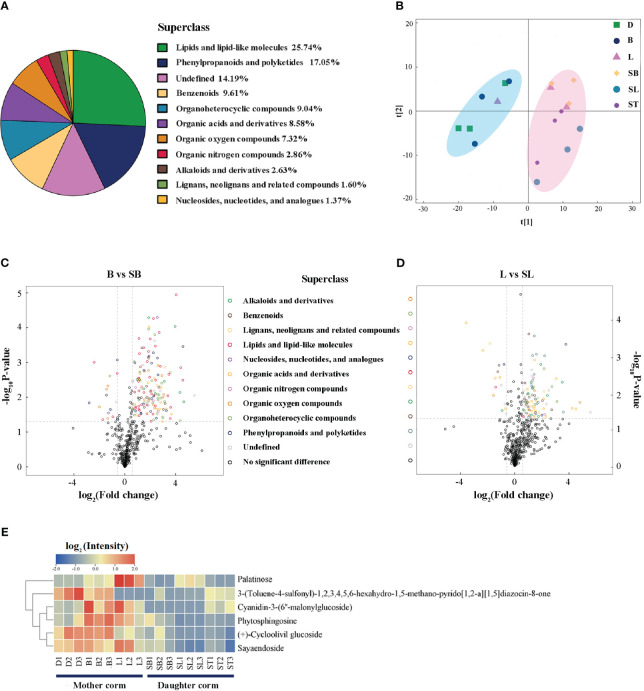
Differential metabolite analysis between mother and daughter corms. **(A)** Overview of the 874 metabolites found in corms, and their classification into classes. **(B)** PCA analysis of corm samples at each developmental stage. **(C, D)** Volcano map of differential metabolites between mother and daughter corms. **(E)** The common differential metabolites of the mother corm at bud emergence and leaf maturation compared to the corresponding period of the daughter corm. D, dormant corms; B, bud sprouting stage; L, first leaf maturation stage; SB, second bud sprouting stage; SL, second leaf maturation stage; ST, lodging period.

We performed an untargeted metabolite analysis of corms during the growth cycle of *A. muelleri*. A total of 874 metabolites were identified (**Table S4**), and the percentage of each class of metabolite is shown in **Figure 3**. The percentage of organic oxygen compounds in which carbohydrates were located was 7.32%. From the results of principal component analysis (PCA), the mother and daughter corms were separated by PC1, except for L (the period when daughter corms start to grow and are present at the same time as the mother corms). This implies a clear difference in the metabolites accumulated in the mother and daughter corms. In addition, there may be a partial interchange of metabolites between the mother and daughter corms during the early growth stages of the daughter corm.

From the differentially accumulated metabolites between mother and daughter corms, more than 80.77% of the differential metabolites (molecular mass< 1.5 kDa) tended to accumulate in the mother corms (**Figures 3C, D**). For example, 29 metabolites were identified to differ between B (in the mother corm) and SB (in the daughter corm), and 25 of these metabolites accumulated mostly in the mother corm. The amount of glycoside pigment, cyanidin-3-(6’’-malonylglucoside), was 44.33 times greater in mother corms compared to daughter corms (**Table S5**). In addition, a small number of metabolites had higher levels in the daughter corms, e.g., 1-methylguanosine, which belongs to nucleosides, exhibited 2.43 times higher amounts.

Six differentially accumulated metabolites were shared between B vs. SB and L vs. SL: two carbohydrates, three glycosides, and one alkaloid (**Figure 3E**). Although these metabolites accumulated in higher amounts in the mother corms compared to daughter corms, there were differences in their accumulation patterns. The content of two carbohydrates increased with leaf development, which we hypothesized to be due to the storage of carbohydrates in the mother corm. There were also three glycosides, sayaendoside, a lignin glycoside, a pigment glycoside, and an alkaloid, all of which accumulated in higher amounts throughout the developmental period of the mother corm. Additionally, as these substances may be involved in the detoxification of the plant, as well as the defense against herbivores (Boeckler et al., 2011; Ali et al., 2019), we hypothesized that the accumulation of these metabolites in the mother corm may be related to stress tolerance.

### 3.3 Comparison of carbohydrate accumulation strategies of mother and daughter corms

**Figure 4:**
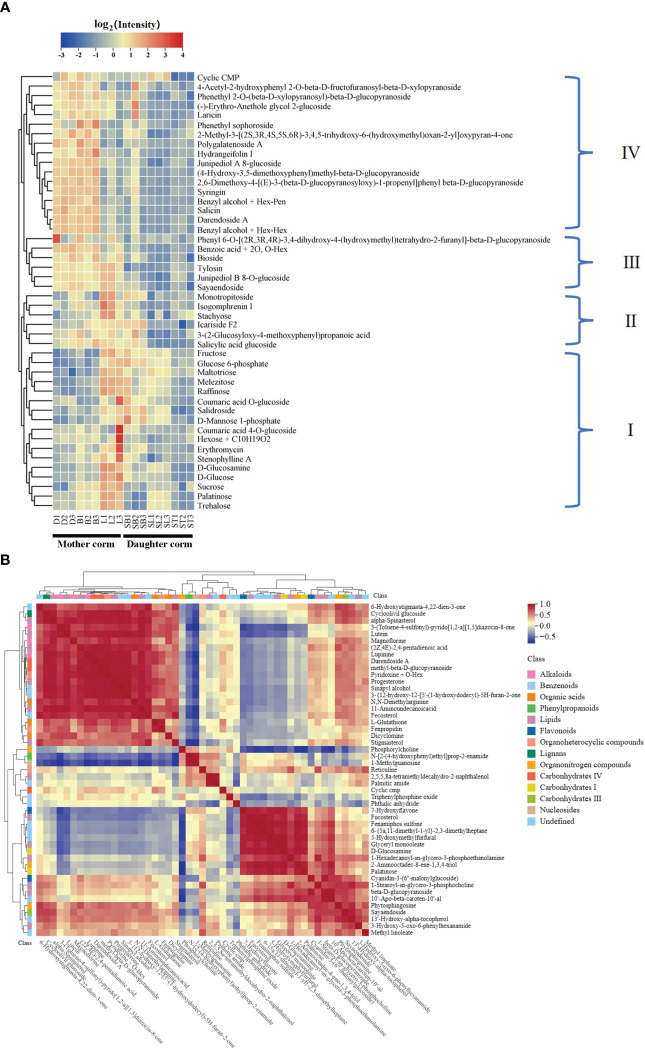
Heat maps showing **(A)** the clustering of carbohydrates and **(B)** correlation analysis with other differential metabolites between daughter and mother corms.

In total, 46 carbohydrates and their conjugates were detected (**Figure 4A**, **Table S6**): nine sugars, consisting of monosaccharides, disaccharides, and some polysaccharides with small molecular weights, and 37 glycosidic substances such as glucose phosphate, sugar alcohols, and glucosamine. These carbohydrates clustered into four branches based on their accumulation patterns in the corms during different developmental periods. Carbohydrates in branch I accumulate mainly in the mother corms at the L stage and in daughter corms at the SL stage. Those in branch II tend to accumulate in the mother corms at the L stage and in daughter corms at the SB stage. Carbohydrates in branch III show high accumulation throughout the developmental period of the mother corms, whereas those in branch IV accumulate mainly in mother corms during dormancy and germination.

There is a clear difference between the metabolites accumulated in the mother and daughter corms. This implies that the metabolites of the corms are not simply replicated during the turnover but undergo a process of material-energy transformation. For example, the accumulation strategies of sucrose and glucose in carbohydrate branch I are quite different in the mother and daughter corms. Sucrose is a major product of photosynthesis in green leaves and accounts for most of the CO_2_ fixed during photosynthesis (Niyogi et al., 2015). It also serves as the principal long‐distance transport compound in most plants and as a storage compound in some plants (Lemoine et al., 2013; Julius et al., 2017). Glucose is an important monosaccharide that provides energy to plants (Siddiqui et al., 2020). These carbohydrates gradually accumulated in the mother corms during the development of the first leaf, suggesting that they act as carbon reservoirs in the mother corms. However, the sucrose and glucose contents decreased significantly with the emergence of the second leaf, and although the accumulation of these sugars in the daughter corm was slightly elevated at the second leaf maturation stage, it was still significantly lower than that in mother corms. This suggests that these substances may be used as carbon suppliers rather than reservoirs in daughter corms.

Correlation analysis of carbohydrates and other differential metabolites in the mother and daughter corms also proved that the accumulation patterns of each group of carbohydrates were not identical (**Figure 4B**). Flavonoids with antifungal activity were significantly positively correlated with carbohydrates in branch I but negatively correlated with those in branch IV. Alkaloids, which have a defense function against microorganisms, co-accumulated more consistently with the carbohydrates in branch IV. Together, these results indicate that the carbohydrates of each branch perform different functions in the daughter and mother corms. The amount of carbohydrates in branch IV was higher in the dormant and sprouting stages of the mother corm and decreased with leaf development, indicating that this type of carbohydrate provides a source of carbon and energy for plant growth and development. This is quite different from the pattern of the branch I carbohydrates mentioned above. It further confirms that these metabolites have different accumulation strategies in the daughter and mother corms of *A. muelleri*.

### 3.4 Enrichment analysis of KEGG pathway for differential metabolites and differentially expressed genes between daughter and mother corms

**Figure 5:**
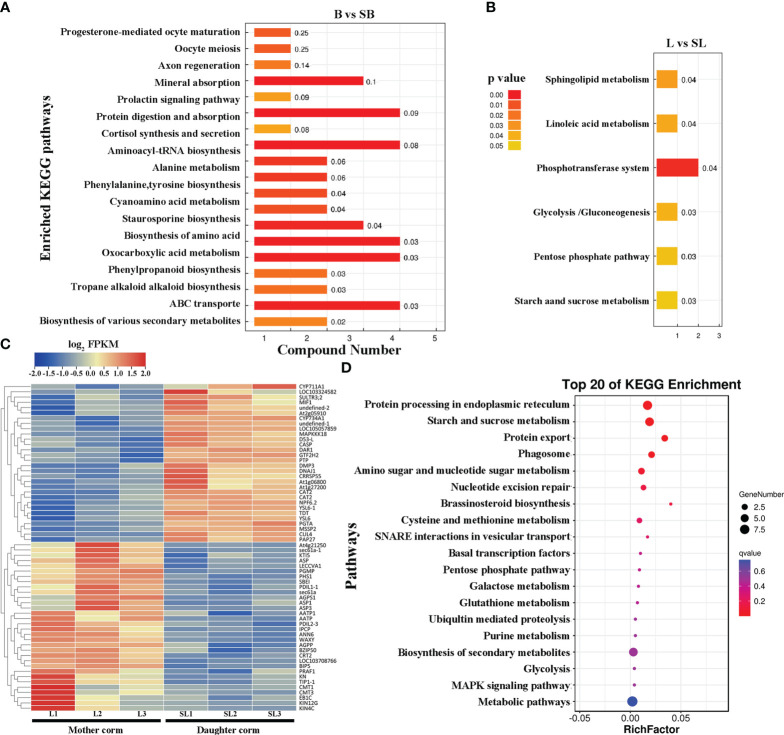
KEGG pathway enrichment analysis for **(A, B)** differential metabolites and **(C, D)** differentially expressed genes between daughter and mother corms of *A muelleri*. B, bud sprouting stage; L, first leaf maturation stage; SB, second bud sprouting stage; SL, second leaf maturation stage.

We performed a KEGG pathway enrichment analysis for differential metabolites between the daughter and mother corms. Metabolites that differed between B and SB (**Figure 5A**) were mainly involved in the synthesis and uptake of amino acids and proteins, as well as transmembrane transport. When comparing L and SL (**Figure 5B**), the differential metabolites were mainly involved in the glycolysis, pentose phosphate pathway, and starch and sucrose synthesis, which are closely related to carbohydrate metabolism. To further elucidate the regulatory mechanism of differential metabolite accumulation during the replacement of mother and daughter corms, the differentially expressed genes between mother corms at L and daughter corms at SL were investigated (**Figure 5C**). These differentially expressed genes were mainly involved in protein processing pathways in the endoplasmic reticulum and starch and sucrose metabolism (**Figure 5D**). Combined with the metabolomic results, this indicates that carbohydrate metabolism pathways may play a key role in the corms turnover process of *A. muelleri*.

### 3.5 Major carbohydrates and genes involved in starch, sucrose, and glucomannan synthesis pathways

Plants specialize in harnessing light energy to convert simple nutrients such as CO_2_, water, and inorganic ions into carbohydrates required for their autotrophic lifestyle (Niyogi et al., 2015). Some of the carbon assimilated by the leaves through photosynthesis is immediately used for plant growth, whereas the remainder is stored in several forms, including vacuolar sucrose or fructose (Collucci et al., 2019); however, the most common storage form is starch (Smith et al., 2005; Smith and Stitt, 2007; Dennis and Blakeley, 2015). The konjac corm is an important carbon storage tissue that sustains plant growth when photosynthesis is not possible.

**Figure 6:**
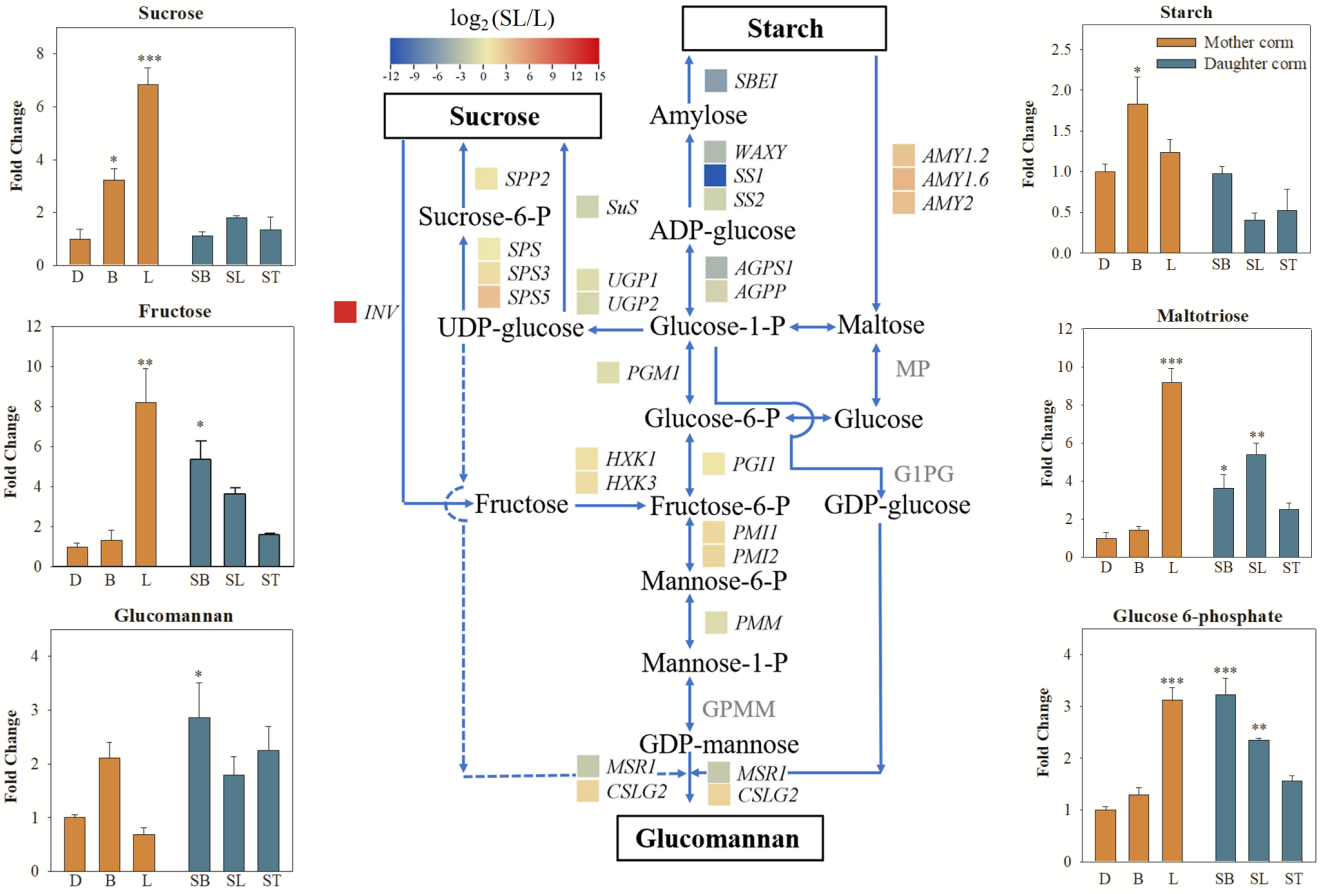
Comparison of the major carbohydrates and genes involved in starch, sucrose, and glucomannan metabolism pathways compared between daughter and mother corms. Changes in transcript levels are indicated by color codes. Red shows upregulated, and blue indicates downregulated gene expression (using FPKM values) in daughter corms at the second leaf maturity period (SL) compared to mother corms at the first leaf maturity period (L). The values of the fold change (SL vs. L) are log_2_ scaled. The pathways connecting sucrose, starch and glucomannan are shown. Dash lines represent speculative pathways. The bar charts show the changes in the carbohydrates content of daughter and mother corms at different developmental stages. The values represent means ± SE and are fold change in content of daughter corms compared to the content in mother corms at the dormant stage **(D)**. Asterisks indicate statistical significance using One-way ANOVA with Tukey test (*: P< 0.05; **: P< 0.01; ***: P< 0.001). D, dormant corms; B, bud sprouting stage; L, first leaf maturation stage; SB, second bud sprouting stage; SL, second leaf maturation stage; ST, lodging period; SBE, starch branching enzyme; WAXY, granule-bound starch synthase; SS, starch synthase; AGPS, ADP-glucose pyrophosphorylase; AGPP, Nucleotidyl transferase PGM, phosphoglucomutase; PGI, glucose-6-phosphate isomerase; PMI, mannose-6-phosphate isomerase; PMM, phosphomannomutase; GMPP, GDP-mannose pyrophosphorylase; MSR, mannan-synthesis related; CSLG, cellulose synthase-like protein G; AMY, alpha-amylase; MP, maltose phosphorylase; G1PG, glucose-1-phosphate guanylyl transferase; Sus, Sucrose synthase; INV, invertase; SPP, sucrose-phosphatase; SPS, sucrose-phosphate synthase; UGP, UTP-glucose-1-phosphate uridylyltransferase; HXK, hexokinase.

As shown in **Figure 6**, the synthesis of starch was inhibited, whereas the starch hydrolysis pathway was activated in daughter corms at the SL stage compared to mother corms at the L stage. The expression profiles of the involved genes are presented in **Table S7**. The FC value of the starch synthesis gene, *AkWAXY*, was only 0.096 (SL vs. L), which was less than one-tenth of that in the mother corms. In contrast, the FC values (SL vs. L) of *AMY1.2*, *AMY1.6*, and *AMY2*, which encode alpha-amylase that promotes the hydrolysis of starch to maltose, were 7.88, 15.63, and 9.62, respectively. Although several genes in the sucrose synthesis pathway were upregulated to some extent, the gene encoding invertase (*INV*), a key enzyme in the sucrose catabolic pathway, was drastically upregulated in the daughter corm (FC = 351.78). This suggests that sucrose and starch are not the major storage carbohydrates in daughter corms.

In daughter corms, starch is hydrolyzed to maltose, which is further converted into hexose phosphate before entering the hexose phosphate pool. The hexose phosphate pool consists of three metabolic intermediates: glucose 6‐phosphate (G6P), glucose 1‐phosphate (G1P), and fructose 6‐phosphate (F6P). These three metabolites can be interconverted, allowing the plant to monitor and balance sucrose and starch levels (Fettke et al., 2008; Kunz et al., 2014). The levels of G6P were significantly elevated in the daughter corm, leading to the activation of sucrose‐phosphate synthase (SPS), which is thought to promote sucrose synthesis (Heldt and Piechulla, 2021). However, the results showed that despite the upregulation of SPS expression, sucrose content remained low in daughter corms. This may be due to a significant increase in the expression of *INV*. Sucrose catabolism is closely related to polysaccharide synthesis (Babb and Haigler, 2001; Ruan, 2014). Fructose produced by sucrose catabolism can be converted to mannose, which can be condensed to glucomannan, catalyzed by cellulose synthase-like protein G2 (CSLG2), with GDP-glucose converted from G1P in the hexose phosphate pool (Yu et al., 2014; Gao et al., 2022). Here, the expression of *CSLG2* in daughter corms was 3.85 times higher than that in mother corms. The RT-qPCR results for genes in the sucrose, starch, and glucomannan metabolic pathways were generally consistent with the trends in transcriptome data, indicating that the reliability of the transcript data was high.

Given the significantly higher KGM content of the daughter corms compared to that of mother corms, we hypothesized that the catabolism of sucrose and starch promotes carbon flow into the hexose phosphate pool. When the levels of the glucomannan precursors, G1P and F6P, increased, the glucomannan synthesis enzyme CSLG2 was activated, resulting in elevated glucomannan content in daughter corms. Natural polysaccharides are synthesized to fulfill many different functions, the high glucomannan content in daughter corms may not only serve as an energy reserve but also helps to provide structural support and maintain cellular homeostasis (Chylińska et al., 2017; Torres et al., 2019), and we hypothesized that this property would be helpful in coping with the drying and low-temperature conditions of the forthcoming dormant period.

## 4 Conclusion

Carbohydrates that are transported from source to sink have been proposed to play an important role in regulating the formation of daughter corms in konjac. In this study, we found that daughter corms tend to accumulate KGM rather than starch and sucrose, indicating that the carbohydrate utilization strategy differs between daughter and mother corms. KGM has significant applications in medical, agricultural, chemical, and other industries. Therefore, unraveling the molecular mechanisms underlying the process of carbohydrate accumulation in corm will contribute to improving the quality of konjac.

## Data availability statement

The datasets presented in this study can be found in online repositories. The names of the repository/repositories and accession number(s) can be found in the article/**Supplementary Material**.

## Author contributions

YQ: Data curation, Formal Analysis, Writing – original draft, Writing – review & editing, Funding acquisition. PG: Writing – review & editing, Data curation, Formal Analysis, Software. SY: Writing – review & editing, Data curation, Formal Analysis. LL: Writing – review & editing, Software, Data curation. YK: Writing – review & editing, Methodology, Visualization. HW: Writing – review & editing, Validation, Visualization. FH: Writing – review & editing, Conceptualization, Data curation, Supervision. LY: Supervision, Writing – review & editing, Funding acquisition, Conceptualization.

## Supplementary material

Supplementary Table 1 The quality and concentration of the extracted RNA.

Supplementary Table 2 Sequences of primers used in RT-qPCR.

Supplementary Table 3 Growth traits during the growth cycle of *A. muelleri*.

Supplementary Table 4 Untargeted metabolite analysis of corms during the growth cycle of *A. muelleri*.

Supplementary Table 5 Differential metabolite analysis between mother and daughter corms.

Supplementary Table 6 Carbohydrates measured in corms during the growth cycle of *A. muelleri*.

Supplementary Table 7 The expression of major genes involved in glucomannan metabolism pathways in corms of *A. muelleri*.
